# Lymphomatoid Granulomatosis of Central Nervous System and Lung Driven by Epstein Barr Virus Proliferation: Successful Treatment with Rituximab-Containing Chemotherapy

**DOI:** 10.4084/MJHID.2014.017

**Published:** 2014-02-16

**Authors:** Ruben Fernandez-Alvarez, ME Gonzalez, Almudena Fernandez, AP Gonzalez-Rodriguez, JM Sancho, Francisco Dominguez, Carmen Fernandez

**Affiliations:** 1 Department of Hematology, Hospital de Cabueñes, Gijon, Spain.; 2 Department of Pathology, Hospital de Cabueñes, Gijon, Spain.; 3 Stem Cell Transplant Unit, Hospital Universitario Central de Asturias, Oviedo, Spain.; 4 Clinical Hematology Department, ICO-Hospital Germans Trias i Pujol, Barcelona, Spain.

## Abstract

Lymphomatoid granulomatosis (LYG) is a very rare Epstein-Barr virus (EBV) associated B-cell lymphoproliferative disorder. We report the case of a 41-year-old man who presented with fever and respiratory symptoms. Computed tomography showed multiple nodules in both lung fields. Polymerase chain reaction (PCR) analysis for EBV was positive in bronchoalveolar lavage and biopsy of lung node yielded a diagnosis of LYG, grade III. Shortly after initiation of treatment with agressive chemotherapy, neurological deterioration appeared. Neuroimaging findings revealed hydrocephalus and PCR analysis of the cerebrospinal fluid (CSF) was positive for EBV. Treatment with intravenous rituximab led to rapid reduction of EBV load in CSF, along with clinical and radiological improvement. After completion of treatment with immunochemotherapy, an autologous stem cell transplantation was performed. Patient stays in remission 18 months after diagnosis.

## Introduction

Lymphomatoid granulomatosis (LYG) is a rare B-cell lymphoproliferative disorder characterized by an angiocentric and angiodestructive lymphoid proliferation. Cytological composition consists of large atypical B cells (which are Epstein-Barr [EBV]-positive) accompanied by reactive T cells.^1^ Progression to a diffuse large B-cell lymphoma (DLBCL) occurs in up to 15% of cases,^2^ and distinction between high grade LYG and lymphoma can be subtle.

Clinical presentation is heterogeneous. Pulmonary involvement is almost always present, while other common sites include central nervous system (CNS) and skin.^2^

Currently, LYG is considered an EBV related B-lymphoproliferative disorder,^3^ similar to other lymphoproliferative disorders such as post-transplantation lymphoproliferative disease. Although LYG is most commonly diagnosed in patients without over immunodeficiency, it is probable that most cases display an immunologic defect that is unable to adequately control EBV-infected B-cells.^4^

Disease course is unpredictable and there is no clearly established treatment. After the recognition of EBV-positive B cells involvement, rituximab has been considered as a promising treatment.^5^

We report here a case of an agressive LYG involving skin, lungs and CNS, with documented active EBV replication, and effective treatment with chemotherapy plus rituximab.

## Case Report

A 41-year-old male presented with fever, weight loss and cough. Physical examination revealed multiple erythematous plaque lesions on his back. Laboratory results revealed elevated serum lactate dehydrogenase (LDH) level, mild anemia and elevated C reactive protein level. Computed tomography (CT) scan showed ground glass infiltrates at lower lobes. Antibiotics were started, without improvement. Serologic tests for Mycoplasma, hepatitis B, hepatitis C and Human Immunodeficiency virus (HIV) were negative, whereas Cytomegalovirus (CMV) and EBV were positive, indicating previous infections. Additional workup showed negative antinuclear antibodies and antineutrophil cytoplasmic antibodies (ANCA). Two skin biopsies were performed, showing a dense T-cell lymphoid infiltrate, EBV-ISH negative. Steroid treatment was started (1 mg/kg/day), and fever and skin lesions disappeared rapidly. However, patient developed confusion, gait imbalance and diplopia.

**Figure 1:**
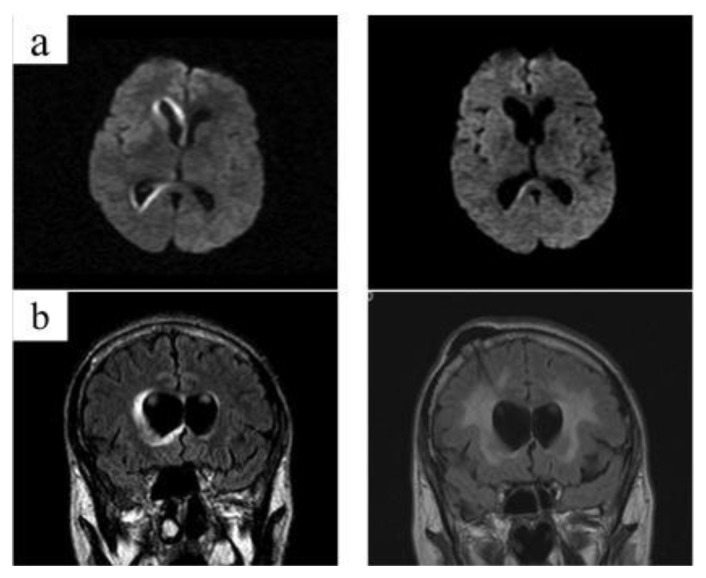
Cranial magnetic resonance imaging (MRI) findings prior to (left column) and after (right column) rituximab-containing chemotherapy. At presentation, MRI diffusion sequence (a) and coronal T2-FLAIR sequence (b) show hyperintense lesions in the periventricular withe matter (predominantly around right lateral ventricle). Follow-up MRI after completion of 2 courses of immunochemotherapy demonstrates marked regression of hyper-intensities (right column). Note Ommaya reservoir placed in right lateral ventricle (b, right column).

Cerebrospinal fluid (CSF) analysis revealed: 10 white blood cells (WBC, all mononuclear), protein 1.11 g/L, and glucose 71 mg/dl. CSF flow cytometry detected a polyclonal T-cell population. Cranial magnetic resonance imaging (MRI) showed diffuse hyperintense lesions involving white matter around ventricles, without contrast enhancement (Figure 1[a]).

**Figure 2:**
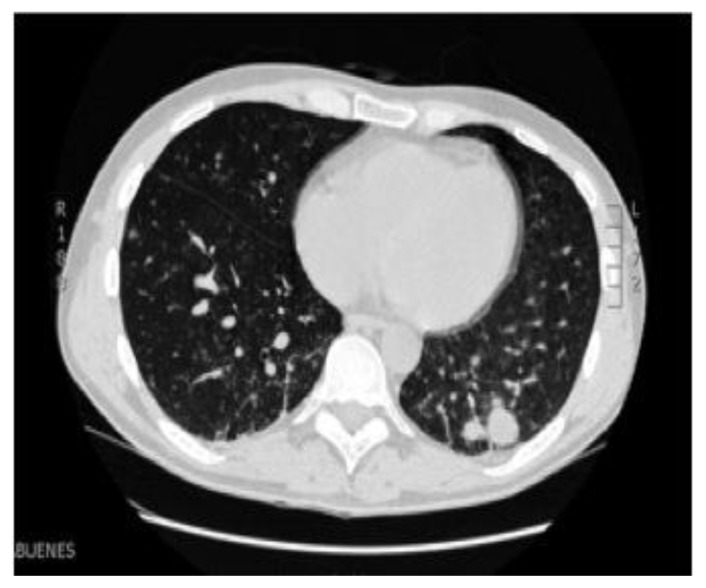
Computed tomography show pulmonary nodular lesions in middle and lower lobes.

**Figure 3:**
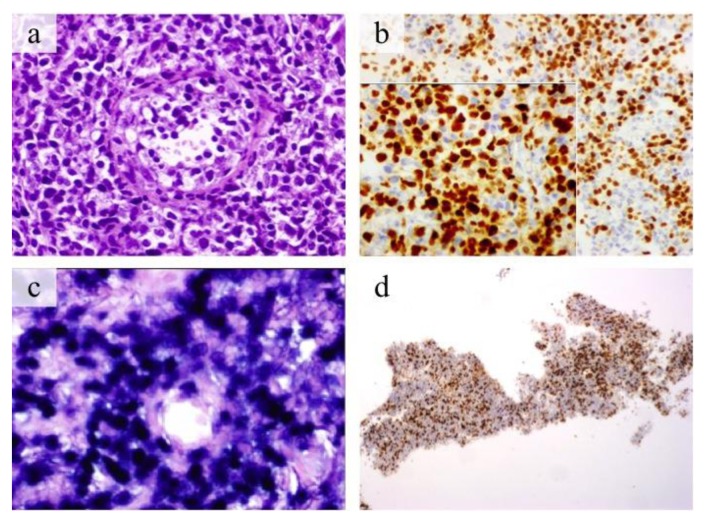
Histological examination of lung node biopsy reveals a dense lymphoid infiltrate with an angiocentric distribution, showing transmural infiltration (a). Immunohistochemistry reveals these cells express B-cell markers (MUM1 + [b]) and are strongly EBV-ISH positive (more than 50 high-power fields) (c). All these findings are consistent with LYG, grade III. Extensive T cell infiltration accompanying is also observed (CD3-positive cells [d]). EBV: Epstein-Barr virus

Two weeks later, respiratory symptoms worsened and a second CT revealed multiple pulmonary nodules in middle and lower fields, some of them with cavitation (Figure 2). Microbiological tests of bronchoalveolar lavage (BAL) were negative, except amplification of EBV-DNA by polymerase chain reaction (PCR), that yielded 4080 copies/mL. Biopsy of lung node showed a highly polymorphic infiltrate of large and atypical lymphoid cells, displaying an angiocentric and angiodestructive distribution (Figure 3[a]). Immunostains showed B-cell proliferation (PAX-5+, MUM-1+ -Figure 3[b]-) with very intense EBER expression (Figure 3[c]), together with T-cell proliferation (Figure 3[d]). These findings led to suspicion of T-cell lymphoma, but after later review, final diagnosis was LYG, grade III. Complete staging revealed mild hepatosplenomegaly, while bone marrow was normal.

**Figure 4:**
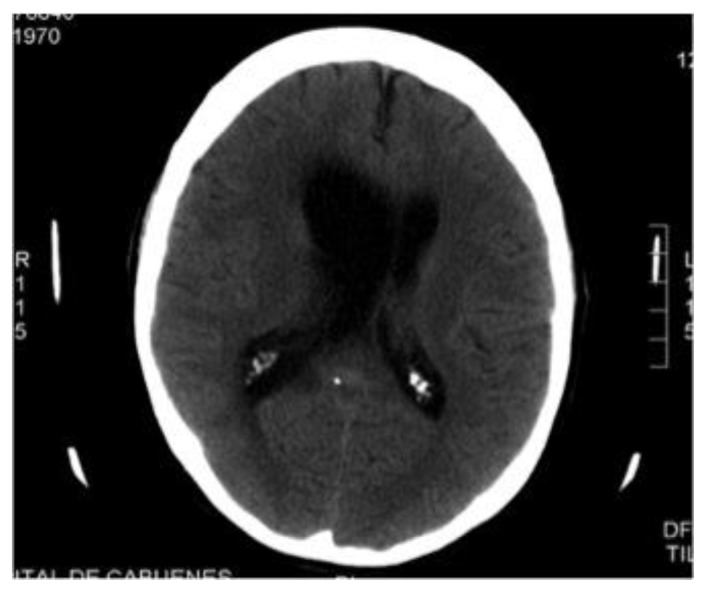
Cranial computed tomography reveals marked hydrocephalus and hipodensity around ventricles.

Because of suspicion of T-cell lymphoma with probable CNS involvement, treatment was started with a high-dose methotrexate (MTX) based protocol (ifosfamide, 1500 mg/m^2^ daily for 5 days plus mesna; etoposide, 150 mg/m^2^ daily for 3 days; cytarabine, 100 mg/m^2^ daily for 3 days; and MTX, 3 g/m^2^ on Day 5, with leucovorin rescue) plus intrathecal application of MTX, Ara-C, and hydrocortisone. Shortly after, the patient developed somnolence, incoherent speech, and nystagmus. Cranial CT scan revealed important hydrocephalus with worsening of previous findings (Figure 4). An Ommaya reservoir was placed and examination of CSF showed a WBC count of 8cells/mL, with normal glucose and protein levels. The Gram staining and culture were negative. PCR analysis for EBV was positive, yielding 43700 copies/mL. HHV-6, CMV, HSV, VZV and JCV were negative by PCR assay.

**Figure 5:**
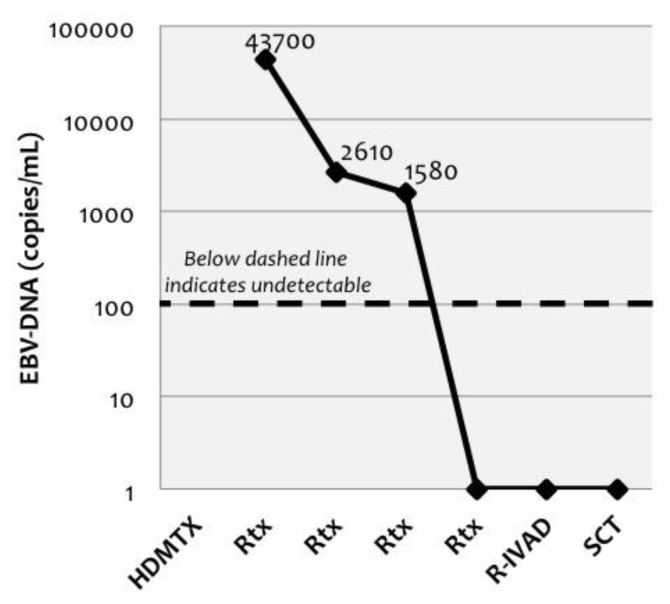
Changes in EBV-DNA copies in cerebrospinal fluid (CSF) during clinical course of the patient, according to administration of courses of chemotherapy and rituximab. EBV: Epstein-Barr virus

At this stage, treatment with rituximab (375 mg/m^2^/iv) was added, once weekly for 4 doses. EBV-DNA copies in CSF rapidly declined in the following weeks (Figure 5). A cranial MRI performed after completion of rituximab demonstrated significant improvement, and pulmonary CT scan showed decreasing size of lung nodules.

Once patient improved, he received one further cycle of the same high-dose MTX-protocol, together with rituximab and intrathecal chemotherapy. Autologous stem cell transplantation was scheduled. The preparative regimen consisted of total body irradiation (10 Gy) and cyclophosphamide (100 mg/Kg) without major complications. On follow-up, both cerebral MRI and pulmonary CT scan have not shown progression. 18 months after the diagnosis, patient remains in remission.

## Discussion

LYG was first described by Liebow et al.^6^ in 1972, and originally was considered a form of T-cell lymphoma, based on the predominant T-cell infiltrate. It was later demonstrated that LYG is an EBV-associated B-cell lymphoproliferative disorder, accompanied by a large number of reactive T-cells.^7^ The distinctive morphologic feature of LYG is an angiocentric and angiodestructive lymphoid infiltrate, with infiltration of the vascular wall and variable degree of necrosis. Currently, LYG is considered a lymphoproliferative disorder with a broad pathological and clinical spectrum. Lipford et al.^8^ classified LYG lesions into 3 histologic grades on the basis of the degree of cytologic atypia and necrosis. The number of EBV-positive cells correlates with histologic grade.^1^ In grade 1 lesions, EBV-positive cells are infrequent whereas they are readily detected in grade 2 and 3 lesions. It is important to note that the number of EBV-cells may vary over time or between sites, as reflected in our case (skin, grade 1; lungs, grade 3).

Clinically, LYG demonstrates predilection for men, most often in adults between the 4^th^ and 6^th^ decades of life. The lungs are virtually always affected in LYG.^2^ Radiologically, pulmonary lesions are heterogeneous, from diffuse infiltrates to nodules.^9^ According to its angiodestructive nature, occasional cavitation/necrosis is seen, as in our case.

Skin is affected in approximately 45% of patients. Cutaneous lesions may appear before the pulmonary disorder,^10^ as in our case. Dermatologic manifestations are diverse, but most typically appear as nodules. The clinical pattern of the cutaneous lesions correlates with histological grade. Beaty et al.^10^ showed that the plaque lesions were negative for EBV and demonstrated IgH polyclonal patterns. In contrast, angiodestruction, cellular atypia and EBV positivity are more common in nodules. Our case presented erythematous plaques, without EBV, which is consistent with grade 1 LYG.

Central nervous system (CNS) involvement occurs in 25–35% of cases, usually accompanying pulmonary lesions.^2^ Neuroimaging findings are variable. Mizuno et al.^11^ reviewed 45 LYG cases involving CNS, and classified them as tumorous lesions and non-tumorous lesions (multiple focal lesions or diffuse involvement, as in our case). Most common finding consists of multiple focal lesions involving the white matter, deep grey matter, or the brainstem,^12^ showing high signal intensity (on T2-weighted images) and contrast enhancement; however it’s possible that some lesions only manifest T2 prolongation. Our case presented diffuse involvement of white matter, with typical T2 hyperintensity, but did not show contrast enhancement. Histologic documentation of brain lesions is not usually available and CSF analysis presents low sensitivity for the diagnosis of LYG in the brain,^12^ as in our case. High EBV-DNA levels were found in our case, which is consistent with the biology of the process. No previous reports exist on the detection of EBV-DNA in CSF of patients with cerebral LYG, so significance of this finding is unknown. We think measurement could be clinically useful for diagnosis support and for monitoring treatment response. In our case, virologic response in CSF correlated with radiological and clinical response.

LYG is considered an EBV driven process. Its implication was first described in 1990 when EBV DNA was found in tissue samples of 21 out of 29 patients with LYG.^3^. Evidence of subtle immune deficits has been found in otherwise immunocompetent individuals with the disease.^4^ Disease hypothesis suggests LYG arise in the setting of a dysregulation of EBV surveillance (deficit of EBV-specific CD8 T-cells). Progressive oncogenic events transform lower grade to higher grade disease: grades 1 and 2 are polyclonal or oligoclonal and immune dependent; grade 3 disease is monoclonal and immune independent.^13^

Therapy of LYG includes corticosteroids, chemotherapy or observation, generally with poor outcomes,^14^ as these therapies can potentially worsen the intrinsic immunologic defect. In our case, neurological condition deteriorated after steroids and chemotherapy. We hypothesized that decline in cellular immunity caused an increase of EBV infected cells, as we found a strikingly high viral load of EBV in CSF.

A risk-adapted treatment strategy has been developed by the US National Cancer Institute (NCI),^13^ with the objective of improving immunologic defect and eradicating EBV-positive B cells. They use interpheron alpha for low-grade LYG, and immunochemotherapy (rituximab plus CHOEP regimen) for high-grade LYG.

Rituximab is considered a promising treatment for LYG, after the recognition of EBV-positive B-cells involvement. Recently, Hernandez et al.^5^ reviewed the use of rituximab in LYG. To date, a total of 22 cases have been reported: 11 patients had improvement (6 in monotherapy, 5 in combination with other drugs), whereas 7 patients progressed. The role of rituximab for CNS lesions is also unclear: of 22 patients reported, only 7 had CNS involvement.^5^,^15^–^19^ Responses were successful in 3 cases, all of them received rituximab as monotherapy.^15^–^17^

In conclusion, our case presented asynchronous manifestations of LYG at skin, lungs and CNS, with detection of EBV-DNA at sites of involvement. Rituximab was effective in the treatment, and we demonstrate that clearance of EBV viral load in CSF correlated with clinical and radiological improvement.
